# Computer Vision Syndrome and Associated Factors among Computer Users in Debre Tabor Town, Northwest Ethiopia

**DOI:** 10.1155/2018/4107590

**Published:** 2018-09-16

**Authors:** Awrajaw Dessie, Fentahun Adane, Ansha Nega, Sintayehu Daba Wami, Daniel Haile Chercos

**Affiliations:** ^1^Department of Environmental and Occupational Health and Safety, Institute of Public Health, University of Gondar, Gondar, Ethiopia; ^2^South Gondar Zonal Health Office, Debre Tabor, Ethiopia; ^3^Public Health Faculty, College of Health Sciences, Addis Ababa University, Addis Ababa, Ethiopia

## Abstract

**Background:**

Globally, computer is one of the common office tools used in various institutions. Using computer for prolonged time led to the users at greater health risk of computer vision syndrome (CVS). Computer vision syndrome is the leading occupational health problem of the twenty-first century. About 70 percent of computer users are suffered from CVS. Besides the health problems, CVS causes inefficiency at workplace and deteriorate quality of work. The problem of CVS and its risk factors are not well known in Ethiopia.

**Method:**

A cross-sectional study was conducted to assess the prevalence of CVS and associated factors among computer user government employees in Debre Tabor town from February to March, 2016. Multistage random sampling method was applied to select 607 study participants, and the data were collected by using a structured questionnaire. Computer vision syndrome was measured by self-reported method. Bivariate and multivariable binary logistic regression analyses were performed using SPSS version 20. Significance level was obtained at 95% CI and *p* value < 0.05.

**Results:**

The prevalence of CVS was 422 (69.5%) with 95% CI of 65.60, 73.0%. Blurred vision, eyestrain, and eye irritation were the commonest reported symptoms of CVS with proportion of 62.60%, 47.63%, and 47.40%, respectively. Occupation: officer (adjusted odds ratio (AOR) = 4.74) and secretary (AOR = 9.17), daily computer usage (AOR: 2.29), and preexisting eye disease (AOR = 3.19) were risk factors for CVS. However, computer users with high payment, who took regular health break, and with good knowledge on computer safety measures were less impacted by CVS.

**Conclusion:**

The prevalence of computer vision syndrome was found to be higher in Debre Tabor town. Monthly income, occupation, daily computer usage, regular health break, knowledge, and preexisting eye disease were predictor variables for CVS. Optimizing exposure time, improving awareness on safety measures, and management support are important to tackle CVS.

## 1. Introduction

Globally, personal computers were one of the commonest office tools. It had become a necessity in the 21st century and has been regularly used in various institutions such as government offices, academic institutions, and banking systems [1]. A continuous use of computer for an extended time causes vision problem called computer vision syndrome [2]. Computer vision syndrome (CVS) is defined by the American Optometric Association as a complex of eye and vision problems related to the activities which stress the near vision and which are experienced in relation to or during the use of computers [3]. It encompasses a group of visual symptoms which crop up from the extended viewing of the digital screen, when the demands of the task exceed the abilities of the viewer. Symptoms of CVS which are referred to as digital eye strain include dry and irritated eyes, eye strain/fatigue, blurred vision, red eyes, burning eyes, excessive tearing, double vision, headache, light/glare sensitivity, slowness in changing focus, and changes in color perception [4].

Computer vision syndrome (CVS) is the leading occupational hazard of the 21st century and its symptoms affect nearly about 70 percent of all computer users [5]. Globally, CVS is one of the major public health problems and reduced productivity at work, increased error rate, reduced job satisfaction, and impaired visual abilities. A worldwide data show nearly 60 million people suffering from CVS and 1 million new cases occurred each year [6]. Given the low availability and utilization of personal protective equipment, the high workload, and the limited break time while using computer in developing countries, the burden of CVS is very high [7].

The public health burden of CVS was becoming the concern of policy makers and attracts the attention of researchers. A study conducted in Abuja, Nigeria, reported that 40% of computer users engaged as security and exchange commissioner has suffered from at least one symptom of CVS [8]. A nationwide study in Sri Lanka reported that more than two-thirds of computer office workers were suffering from CVS [9]. A couple of studies conducted in Gondar, Ethiopia, reported that more than 73% of computer users who are working as secretaries, data processors, and bankers were developing CVS [10, 11].

Duration of computer usage, poor lighting, glare, screen brightness, vision problems, and improper workstation setup are risk factors for CVS [12]. Though there is no evidence that CVS symptoms lead to permanent eye damage on top of visual impairment, it causes inefficiency at workplace. Hence, CVS is growing public health issue that can significantly affect the workers' quality of life and their work productivity [5].

Although many studies have reported the prevalence of CVS and the risk factors such as prolonged computer use and poor postures at workstations, most of them were focused on Western adult subjects [13, 14] and few Asian countries [15–18]. Paucity of information found on the problem of CVS and determinant factors in sub-Saharan African Countries, including Ethiopia. The couple of studies conducted in Ethiopia attempted to determine the prevalence of CVS and associated factors among computer users in academic institutions and financial institution, but their focus was on academic institutions and bank workers [10, 11]. However, these studies are not sufficient to explore the nature of CVS and predictor variables at different groups of computer users.

Over the past 30 years, there has been a great advancement in computer technology. It has become almost an indispensable piece of equipment both at office and at home. It is certain that computer has dramatically benefited the society and makes the working condition easier and producing fast output [19]; however, it does associate with health-related problems [2, 4, 5, 8, 9, 11, 13]. Owing to the technological advancement and growing socioeconomic development observed in the world, the use of computer increased dramatically. Sub-Saharan Africa is not an exception on the rate of computer use; however, the users had inadequate knowledge on safety precautions during use of computer. The standard of computers is also poor and not equipped with protective devices from CVS [20]. Therefore, the aim of this study was to assess the prevalence of computer vision syndrome (CVS) and associated factors among computer users of government office workers in Debre Tabor town, northwest Ethiopia. This study shed light on the adverse effect of computer use and its prevention and control methods among computer users in government offices in Ethiopia.

## 2. Methods and Materials

### 2.1. Study Design and Period

A cross-sectional study design was employed from February to March, 2016.

### 2.2. Study Area and Period

The study was conducted in Debre Tabor town, northwest Ethiopia. Debre Tabor, which is the capital of south Gondar Administrative Zone, Amhara regional state, is located 99 km from the capital city of the regional state and 667 km from Addis Ababa. The town consists of sixty government offices with a total of 2752 computer user employees.

### 2.3. Source and Study Population

All computer users who worked in government institutions in Debre Tabor town were the source population, whereas all workers who were using computer in their day-to-day working life for at least one year were taken as study population [9]. The types of tasks performed by the computer users are word processing, spreadsheet processing, data entry and processing, preparing learning and teaching materials, and reading texts on computer.

### 2.4. Sample Size Determination

The sample size was determined by using single population proportion formula with the following assumptions: margin of error 5%, proportion of CVS 73.9% [10], 95% confidence interval, and design effect of 2 and 10% of nonresponse rate to come up with a sample size of 652 respondents.

### 2.5. Sampling Procedure

A multistage random sampling technique was used to select participants from governmental offices. We have used two stages to select the final study participants in this study. In the first stage, twenty government offices were selected randomly from a total of 60 offices in Debre Tabor town. Then, from each selected office, study subjects were selected proportionally to their size by random sampling technique.

### 2.6. Operational Definition

- *Computer vision syndrome (CVS)*: having the symptoms of computer vision syndrome either intermittently or continuously for at least one week during the last twelve months was defined as computer vision syndrome. Presence of pain in and around the eyes, headache, blurred near vision, blurred distant vision, dry eyes, sore/irritated eyes, red eyes, excessive tearing, double vision, twitching of eyelids, and changes in visualizing colors were assessed as symptoms of CVS in this study. The worker who reported one of the above symptoms was considered as positive for CVS [9, 11, 21, 22].
- *Knowledge*: participants were asked to answer 10 knowledge questions about safety measures of CVS. Graded as having “Good knowledge” if they had answered correctly (≥70%) 7–10 questions and (<70%) 0–6 as “Poor knowledge” [9].
- *Computer users*: workers who use computer for their day-to-day working life.
- *Income*: monthly salary of the study participants was used as proxy to measure their income.

### 2.7. Data Collection Method

A self-administered questionnaires supplemented by observational checklists were used to collect sociodemographic data, symptoms of CVS, details of computer usage, potential risk factors (environmental and behavioral factors), and knowledge of computer users on safety measures of CVS. The data collection was carried out by six optometry BSc degree graduates. Two supervisors were also involved in monitoring data collection and checking the completeness of the questionnaires.

### 2.8. Data Quality Control

Training was given for data collectors and supervisors for 3 days on procedures, techniques, and ways of collecting the data. The tool was pretested among 33 (5% of the sample size) government office workers in Nefas Mewcha town, prior to the actual data collection. Afterwards, the necessary modification on the tool was made.

### 2.9. Data Processing and Analysis

The data were entered using Epi-Info version 7 and analyzed using SPSS statistical package for Windows, version 20.0. All assumptions for binary logistic regression were checked. To determine predictor variables for CVS, binary logistic regression model was fitted and variables significant at *p* value < 0.2 in the bivariable analysis were included in the multivariable analysis. Finally, variables found to be significant at *p* value < 0.05 in the final model were declared as predictor variables. Crude odds ratios (COR) and adjusted odds ratios (AOR) with 95% confidence interval were reported in the result.

### 2.10. Ethical Consideration

Ethical clearance was obtained from the Institutional Review Board of the University of Gondar. The purpose of the study was clearly explained to the study subjects, and their verbal consent was obtained. Confidentiality of the information had been maintained at all levels of the study.

## 3. Results

### 3.1. Sociodemographic Characteristics of Respondents

A total of 607 study participants were included in this study with response rate of 93.1%. The median (interquartile range (IQR)) age of the respondents was 29 years (25–35 years). More than half (335 (55.5%)) of the respondents were male, 345 (56.8%) were married, and 308 (50.7%) participants had monthly salary of >3000 ETB (140.16USD) (Table 1).

### 3.2. Environmental and Behavioral Characteristics

Two hundred sixty-six (43.8%) of the participants worked in their current position for more than 5.7 years and 273 (45.0%) used computer for >4.6 hours per day. Two hundred fourteen (35.3%) of the participants were taking regular break during working time. Of which, their mean (±SD) break time was found to be 24.93 ± 11.76 minutes. More than two-thirds (70.7%) of the participants usually used desktop computers. Nearly two-thirds of the participants (61.6%) used ergonomically comfortable sitting chair and nearly quarter of them (23.6%) reported the brightness of their computer screen was dull. Five hundred forty-four (89.1%) of the participants did not wear eyeglass/spectacle. Their major reported reasons were eyeglass can worsen the symptoms, social unacceptability, and not knowing its importance; feeling uncomfortable while wearing it; not to afford to buy; and not prescribed by doctors. On the other hand, 85 (14%) respondents had previous history of eye illness (Table 2).

### 3.3. Prevalence of Computer Vision Syndrome (CVS)

**Figure 1:**
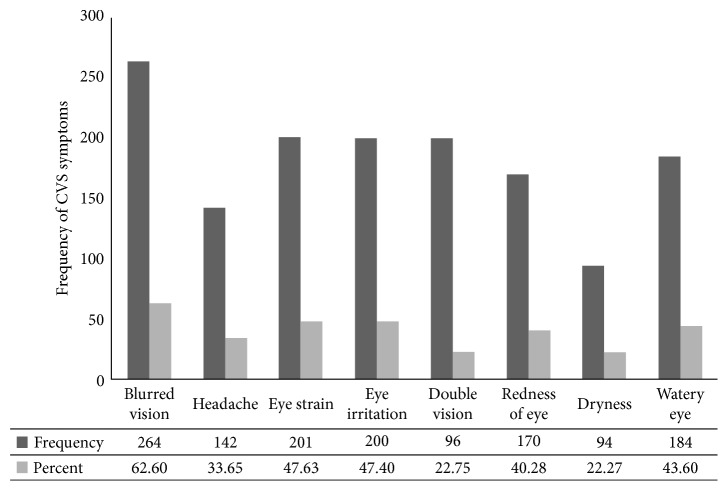
Frequency of computer vision syndrome symptoms among computer users of governmental offices in Debre Tabor Town, Ethiopia (*n*=422).

The self-reported prevalence of computer vision syndrome among computer users was 69.5 % (95% CI; 65.60, 73.0). Blurred vision, eyestrain, and eye irritation were the most common reported symptoms of CVS with prevalence of 62.60%, 47.63%, and 47.40%, respectively (Figure 1).

### 3.4. Factors Associated with Computer Vision Syndrome

The multivariable analysis showed that monthly salary, occupational status, daily computer usage, history of previous eye problem, and knowledge on safety measures of CVS and its adverse effect were found to be determinant factors for CVS.

The odds of developing CVS among computer users who earned a monthly salary in the range of 1500 and 3000 Ethiopian birr (ETB) and greater than 3000 ETB were 74% (AOR = 0.26, 95% CI (0.07, 0.88)) and 89% (AOR = 0.11, 95% CI (0.01, 0.95)) less than computer users who earned less than 1500 ETB. The odds of developing CVS among officers and secretaries were 4.75 (AOR = 4.75, 95% CI (1.77, 12.70)) and 9.17 (AOR = 9.17, 95% CI (2.63, 31.90)) more than the coordinators by occupation.

Participants who used computer for >4.6 hours per day were 2.29 times more likely to develop CVS compared to workers who used computer for 4.6 hours or less (AOR: 2.29, 95% CI (1.43, 3.66)). The study also showed that workers who had previous history of eye illness were 3.19 times more likely to develop CVS than their counterparts. Moreover, workers who had good knowledge on safe use of computer and prevention mechanisms of adverse effect of computer were 42% less likely to develop CVS than their counterparts (AOR: 0.58, 95% CI (0.37, 0.92)). The odds of developing CVS among computer users who regularly adjusted the brightness of their computer screen and who took regular break decreased by 27% (AOR: 0.73, 95% CI (0.58, 0.91)) and 16% (AOR: 0.84, 95% CI (0.53, 0.97)), respectively (Table 3).

## 4. Discussion

This study was aimed at assessing the prevalence of CVS and its predictors. The self-reported prevalence of CVS among Debre Tabor town government office workers was 69.5% (95% CI = 65.60, 73.00). The finding is in line with other studies: 73.9% in University of Gondar, Ethiopia, among secretaries and data processors [10]; 74% in Nigeria [8]; 73% in Gondar, Ethiopia, among bank workers [11]; 74% in Abuja, Nigeria [8]; 67.4% in Sri Lanka among office workers [9]; 72% in Ajman, United Arab Emirates [23]; and 63% in Public University of Putra, Malaysia, among administrative staffs [15]. On the other hand, this study result was less than the findings in Malaysia, which was reported to be 89% [24], and in Chennai, India, which was 80.3% [25]. The possible reason might be either due to the study participants in these areas being university students using computers for a longer time than government office workers or due to students using computers for a long time without eye break for studying rather than office workers who relatively taking most bank workers taking frequent breaks. Regarding the study conducted in India, neck and shoulder pain was included to define CVS, whereas in this study, only ocular and visual symptoms including headache were used to measure CVS. On the other hand, in this study, the eye/visual symptoms which lasted at least 1 week were considered to define CVS, whereas they had no specification on duration of symptoms [25]. These discrepancies might be a possible justification for the reported higher prevalence of CVS in Chennai, India, than our study.

Highly paid computer users were less likely to develop CVS than their low-paid counterparts. This might be due to the fact that high-paid computer users may have greater opportunity to use antiglare and good computers that could reduce the development of CVS. Conversely, low-paid ones were suffering from this disease because they could not afford these facilities. High-paid computer users might have good awareness on computer ergonomics and can optimize safe duration of computer exposure. Income was mentioned as a protective factor for health by different studies [26–28] because high-paid workers can have a better access to health care, which could have alleviated their symptoms. On top of that, these groups were managers and academicians (lecturers and teachers) in this study, who are engaged in less repetitive work such as checking emails and briefly reading notes. A chi-square test shows highly paid computer users took break significantly higher than their counterparts (*X*^2^ = 5.2, *p* value = 0.08), which supported the above argument. Their daily duration of computer use was also significantly less than low-paid computer users (Pearson correlation test between income and computer exposure time: *r* = −0.24, *p* value ≤ 0.001).

This study indicated that officers and secretaries were found to be significantly impacted by CVS compared to managers and coordinators. The possible reason might be officers and secretaries are usually used computer for a long time. A one-way ANOVA and multiple comparison tests confirmed that the daily exposure time of computer was significantly higher among secretaries and officers in this study (*p* value < 0.01).

Daily exposure time was another factor that was statistically significant in this study. Workers who used computers for >4.6 hrs per day were more likely to develop CVS as compared to those who used computers <4.6 hrs (AOR: 2.29, 95% CI (1.43, 3.66)). A computer emits electromagnetic radiation or high-energy blue light, which enables that high energy to stress the ciliary muscle in the eye; ultimately, a prolonged exposure to computer screen led to eye strain. The finding was in line with a study conducted in University of Gondar, Ethiopia [10]. Other similar studies were also reported an increase in the number of hours spent on computer increases the risk of CVS significantly [9, 17, 25, 29, 30]. Hence, reducing the amount of time spent on computer is important to prevent CVS [12].

The odds of developing CVS were higher among computer users who had less frequent or no break. This might be due to the fact that the eyes normally cannot remain focused on the pixel-generated images on a computer screen for a long time, and as such, the eyes must focus and refocus thousands of times by taking frequent breaks for adequate time while viewing the screen, and if the refresh rate is too slow, it causes a high flickering screen, which leads to suffer from symptoms of CVS [11]. The result was in concordance with previous similar studies who reported that taking break is a protective factor for CVS [11, 18, 21, 25]. After working for one hour, taking short breaks for 5 min has been recommended to decrease eye problem without undue influence of work productivity [31].

Previous history of eye illness was found to be significantly associated with CVS (AOR: 3.19, 95% CI (1.49, 6.84)). This finding was supported by a study conducted in Sri Lanka, which indicated preexisting eye diseases were associated with severe CVS [9]. Similarly, a study conducted in India showed that computer users with history of eye problems were at higher risk of developing CVS [32]. Another study in São Paulo, Brazil, showed that headache was high among computer users who worked in poor ergonomic design and lacks adequate eye strain protection mechanisms since sign and symptoms were nonspecific [33]. This might be long-lasting effect of previous illness; the illness may exist till now to feel each other with CVS, lack of care, and treatment related to previous illness, and some of the previous problems are chronic and may exist till now.

Computer users who had good knowledge on safety measures of computer use and its adverse effect were found to be less impacted by CVS (AOR: 0.58, 95% CI (0.37, 0.92)). The result was in agreement with a study conducted in University of Benin, Nigeria [34] and Malaysia [15]. The possible reason might be the workers who have good knowledge are more likely to implement protective measures and will adhere to safe computer use. In general, there is a direct relationship between knowledge and applying safety measures that potentially tackle work-related injuries and diseases. In contrast, a study conducted in Sri Lanka revealed that ergonomics practices knowledge was associated with increased risk of developing CVS [9]. The discrepancy might be in some cases there could be correlation between ergonomics practices knowledge and higher daily computer usage; the later indicated as risk factors for CVS in various studies and the current study.

## 5. Limitation of the Study

The main limitations of this study were ophthalmic examination was not done to measure CVS and the symptoms reported were self-reported. Symptoms that might not be recognized by users would be left unreported. To minimize the unduly effect of self-reported measurement, we have adopted and used standard protocol. Though we have used a protocol that measures CVS symptoms that can be occurred while using computer, some of the symptoms of CVS including blurred vision and eye strain might be caused by uncorrected refractive error that could potentially overestimate the prevalence [35]. According to the current study, the prevalence of CVS among risk groups for refractive error such as aged population and who do not use eyeglass/spectacle was not significantly different compared to their counterparts that show the influence of the bias was not significant. But in the future study, we recommend that the influence of uncorrected refractive error should be addressed methodologically and the measurement of CVS can be supported by ophthalmic examination.

## 6. Conclusion

This study demonstrated that the prevalence of CVS was found to be higher in Debre Tabor town government institutions. Monthly salary, daily exposure time, type of work, and knowledge were the most determinant factors for CVS. Hence, optimizing the exposure time and improving the awareness of users by rigorous training and management support are important to tackle the problem. In the future, it is recommended to determine the additive or synergistic effect of using smartphone and computer tablets on CVS on or off working time.

## Figures and Tables

**Table 1:** Sociodemographic characteristics of computer users in Debre Tabor town, northwest Ethiopia, 2016 (*n*=607).

Variables	Frequency	Percent
Age (years)		
14–29	320	52.7
30–44	258	42.5
45^+^	29	4.8

Sex		
Male	337	55.5
Female	270	44.5

Marital status		
Single	233	38.4
Married	345	56.8
Divorced	17	2.8
Widowed	12	2.0

Monthly salary		
<1500 ETB (70.08 USD)	60	9.9
1500–3000 (70.08–140.16 USD)	239	39.4
>3000 (140.16 USD)	308	50.7

Educational status		
Secondary school complete	7	1.2
College graduate (certificate)	215	35.4
First degree	337	55.5
Second degree	48	7.9

Religion		
Orthodox	565	93.1
Muslim	37	6.1
Protestant	5	0.8

Ethnicity		
Amhara	593	97.7
Oromo	6	1.0
Tigire	8	1.3

Occupation		
Officer	364	60.0
Lecturers and teachers	81	13.3
Secretary	119	19.6
Coordinators and managers	43	7.0

**Table 2:** Behavioral characteristics of computer users and their condition of working environment in Debre Tabor town, northwest Ethiopia (*n*=607).

Variables	Frequency	Percent
Type of computer the workers used		
Desktop only	429	70.7
Both desktop and laptop	108	17.8
Laptop only	70	11.5

Number of working years in the current position		
≤5.7	341	56.2
>5.7	266	43.8

Number of working hours with computer/day		
≤4.6	334	55.0
>4.6	273	45.0

Using ergonomically comfortable sitting chair		
Yes	374	61.6
No	233	38.4

Source of light at the working place		
Natural light	526	86.7
Florescent/light bulb	81	13.3

Brightness of computer screen		
Bright	464	76.4
Dull	143	23.6

Adjusting computer brightness		
Yes	392	64.6
No	215	35.4

Using antiglare for computer screen		
Yes	71	11.7
No	536	88.3

Taking regular break		
Yes	214	35.3
No	393	64.7

Wearing eyeglass at work		
Yes	66	10.9
No	541	89.1

Previous history of eye illness		
Yes	85	14.0
No	522	86.0

Workload on computer		
Yes	213	35.1
No	394	64.9

Knowledge		
Good	345	56.8
Poor	262	43.2

**Table 3:** Multivariable analysis of predictors for computer vision syndrome symptoms among computer users of governmental offices in Debre Tabor town, Ethiopia (*n*=607).

Variables	CVS		COR (95% CI)	AOR (95% CI)
	Yes	No		
Monthly income (ETB)				
<1500	52	8	1.00	1.00
1500–3000	168	71	0.36 (0.16,0.81)^*∗∗*^	0.26 (0.07,0.88)^*∗∗*^
>3000	202	106	0.29 (0.13,0.64)^*∗∗*^	0.11 (0.01, 0.95)^*∗∗*^

Occupation				
Officer	244	120	1.94 (1.03, 3.67)^*∗∗*^	4.74 (1.77,12.70)^*∗*^
Lecturer and teacher	55	26	2.02 (0.95, 4.31)	2.29 (0.90, 5.85)
Secretary	101	18	5.36 (2.45, 11.69)^*∗∗*^	9.17 (2.63,31.90)^*∗∗*^
Coordinators and managers	22	21	1.00	1.00

Number of years in the current position				
≤5.7	247	94	1.00	1.00
>5.7	175	91	0.73 (0.52, 1.04)	0.74 (0.47,1.16)

Number of working hours with computer/day				
≤4.6	196	138	1.00	1.00
>4.6	226	47	3.39 (2.31, 4.96)^*∗∗*^	2.29 (1.43, 3.66)^*∗∗*^

Comfortable computer light				
Yes	242	124	1.00	1.00
No	180	61	1.51 (1.05, 2.17)^*∗*^	1.25 (0.820, 1.89)

Using ergonomically comfortable chair				
Yes	271	103	1.00	1.00
No	151	82	0.70 (0.49, 0.99)^*∗∗*^	0.99 (0.64, 1.54)

Brightness of computer screen				
Bright	344	120	1.00	1.00
Dull	78	65	0.42 (0.28, 0.62)^*∗*^	0.64 (0.39,1.06)

Adjusting computer brightness				
Yes	254	138	0.52 (0.35, 0.76)^*∗*^	0.93 (0.58, 1.47)
No	168	47	1.00	1.00

Taking regular break				
Yes	262	131	0.68 (0.47, 0.78)^*∗∗*^	0.84 (0.53, 0.97)^*∗*^
No	160	54	1.00	1.00

Workload on computer				
Yes	175	38	2.74 (1.83, 4.11)^*∗*^	1.36 (0.84, 2.20)
No	247	147	1.00	1.00

Knowledge				
Good	207	138	0.33 (0.23, 0.48)^*∗∗*^	0.58 (0.37, 0.92)^*∗*^
Poor	215	47	1.00	1.00

Previous history of eye illness				
Yes	76	9	4.29 (2.10, 8.78)^*∗∗*^	3.19 (1.49, 6.84)^*∗*^
No	346	176	1.00	1.00

*Note.* 1.00 = reference, ^*∗*^significant at *p* value < 0.05, ^*∗∗*^significant at *p* value < 0.001.

## Data Availability

Data will be made available from the primary author upon request.

## References

[1] Anshel J. (2005). Visual ergonomics handbook. *Computer Vision Syndrome*.

[2] Sen A., Richardson S. (2007). A study of computer-related upper limb discomfort and computer vision syndrome. *Journal of Human Ergology*.

[3] American Optometric Association (1995). *Guide to the Clinical Aspects of Computer Vision Syndrome*.

[4] Gangamma M. P., Poonam, Rajagopala M. (2010). A clinical study on “computer vision syndrome” and its management with Triphala eye drops and Saptamrita Lauha. *AYU (An International Quarterly Journal of Research in Ayurveda)*.

[5] Charpe N. A., Kaushik V. (2009). Computer vision syndrome (CVS): recognition and control in software professionals. *Journal of Human Ecology*.

[6] Wimalasundera S. (2006). Computer vision syndrome. *Galle Medical Journal*.

[7] Tadesse S., Kelaye T., Assefa Y. (2016). Utilization of personal protective equipment and associated factors among textile factory workers at Hawassa Town, Southern Ethiopia. *Journal of Occupational Medicine and Toxicology*.

[8] Akinbinu T. R., Mashalla Y. (2013). Knowledge of computer vision syndrome among computer users in the workplace in Abuja, Nigeria. *Journal of Physiology and Pathophysiology*.

[9] Ranasinghe P., Wathurapatha W., Perera Y. (2016). Computer vision syndrome among computer office workers in a developing country: an evaluation of prevalence and risk factors. *BMC Research Notes*.

[10] Alemayehu M., Nega A., Tegegne E., Mule Y. (2014). Prevalence of self reported computer vision syndrome and associated factors among secretaries and data processors who are working in University of Gondar, Ethiopia. *Journal of Biology, Agriculture and Healthcare*.

[11] Assefa N. L., Weldemichael D. Z., Alemu H. W., Anbesse D. H. (2017). Prevalence and associated factors of computer vision syndrome among bank workers in Gondar City, northwest Ethiopia. *Clinical Optometry*.

[12] Kozeis N. (2009). Impact of computer use on children’s vision. *Hippokratia*.

[13] Rosenfield M. (2011). Computer vision syndrome: a review of ocular causes and potential treatments. *Ophthalmic and Physiological Optics*.

[14] Yan Z., Hu L., Chen H., Lu F. (2008). Computer vision syndrome: a widely spreading but largely unknown epidemic among computer users. *Computers in Human Behavior*.

[15] Zainuddin H., Isa M. M. (2014). Effect of human and technology interaction: computer vision syndrome among administrative staff in a public university. *International Journal of Business, Humanities and Technology*.

[16] Shrestha G. S., Mohamed F. N., Shah D. N. (2011). Visual problems among video display terminal (VDT) users in Nepal. *Journal of Optometry*.

[17] Sharma A., Khera S., Khandekar J. (2006). Computer related health problems among information technology professionals in Delhi. *Indian Journal of Community Medicine*.

[18] Shantakumari N., Eldeeb R., Sreedharan J., Gopal K. (2012). Computer use and vision-related problems amongst students in Ajman UAE. *Headache*.

[19] Loh K., Redd S. (2008). Understanding and preventing computer vision syndrome. *Malaysian Family Physician: The Official Journal of the Academy of Family Physicians of Malaysia*.

[20] Mbuyisa B., Leonard A. ICT adoption in SMEs for the alleviation of poverty.

[21] Noreen K., Batool Z., Fatima T., Zamir T. (2016). Prevalence of computer vision syndrome and its associated risk factors among under graduate medical students. *Pakistan Journal of Ophthalmology*.

[22] Anshel J. (2007). Diagnosing, treating CVS relies on good case history: basic eye care, ergonomics and optical correction are all part of an effective treatment plan for computer vision syndrome. *Primary Care Optometry News*.

[23] Shantakumari N., Eldeeb R., Sreedharan J., Gopal K. (2014). Computer use and vision. related problems among university students in Ajman, United Arab Emirate. *Annals of Medical and Health Sciences Research*.

[24] Reddy S. C., Low C., Lim Y., Low L., Mardina F., Nursaleha M. (2013). Computer vision syndrome: a study of knowledge and practices in university students. *Nepalese Journal of Ophthalmology*.

[25] Logaraj M., Madhupriya V., Hegde S. (2014). Computer vision syndrome and associated factors among medical and engineering students in Chennai. *Annals of Medical and Health Sciences Research*.

[26] Gedefaw M., Takele M., Aychiluhem M., Tarekegn M. (2015). Current status and predictors of diarrhoeal diseases among under-five children in a rapidly growing urban setting: the case of city administration of Bahir Dar, northwest Ethiopia. *Open Journal of Epidemiology*.

[27] Siziya S., Muula A. S., Rudatsikira E. (2009). Diarrhoea and acute respiratory infections prevalence and risk factors among under-five children in Iraq in 2000. *Italian Journal of Pediatrics*.

[28] Root G. P. (2001). Sanitation, community environments, and childhood diarrhoea in rural Zimbabwe. *Journal of Health, Population and Nutrition*.

[29] Rahman Z. A., Sanip S. (2011). Computer user: demographic and computer related factors that predispose user to get computer vision syndrome. *International Journal of Business, Humanities and Technology*.

[30] Rajeev A., Gupta A., Sharma M. (2006). Visual fatigue and computer use among college students. *Indian Journal of Community Medicine*.

[31] Levy B. S. (2005). *Preventing Occupational Disease and Injury*.

[32] Akms A., Alam S., Do M. (2009). Computer vision syndrome. *The ORION Medical Journal*.

[33] Ingrid Becker S., Michelle Katherine A. X., Valéria Mayaly A. O., Ana Carolina R. P., Rodrigo Cappato D. A. (2015). Primary headaches among adolescents and their association with excessive computer use. *Revista Dor*.

[34] Chiemeke S. C., Akhahowa A. E., Akhahowa B. O. Evaluation of vision-related problems amongst computer users: a case study of University of Benin, Nigeria.

[35] Adane F. (2016). *Prevalence of Self Reported Computer Vision Syndrome and Associated Factors among Computer Users of Governmental Offices in Debre Tabor Town, Northwest Ethiopia*.

